# Comparative effectiveness of colonic stenting alone and with neoadjuvant chemotherapy for patients with left-sided obstructive colon cancer: a meta-analysis

**DOI:** 10.1097/JS9.0000000000002779

**Published:** 2025-07-15

**Authors:** Qin Sun, Xinyi Zhang, Jiani You, Yueshan Pang, Zining Luo, Yuhang Liu, Yuquan Chen, Yixin Sun, Zifeng Zhuang, Zhiwu Li, Anan Yu, Tianliang Yao, Ming He, Xu Liu, Yuan Zhang, Yongfu Xiong, Yixing Ren, Jiebin Xie

**Affiliations:** a Affiliated Hospital of North Sichuan Medical College, Nanchong, Sichuan, China; b Peking University, Beijing, China; c Department of Clinical Medicine, North Sichuan Medical College, Nanchong, Sichuan, China; d Department of General Practice, The Second Clinical Medical College of North Sichuan Medical College, Nanchong Central Hospital, Nanchong, Sichuan, China; e Medical Information Research Group, Department of Stomatology, North Sichuan Medical College, Nanchong, Sichuan, China; f School of Public Health and Preventive Medicine, Faculty of Medicine, Nursing & Health Sciences, Monash University, Melbourne, Victoria, Australia; g First Hospital, Peking University, Beijing, China; h Jinan University, Guangzhou, Guangdong, China; i Beijing Institute of Petrochemical Technology, Beijing, China; j Department of Control Science and Engineering, College of Electronics and Information Engineering, Tongji University, Shanghai, China; k Department of Electronic Engineering, Faculty of Engineering, The Chinese University of Hong Kong, Hong Kong SAR, China; l Department of Gastrointestinal Surgery, Affiliated Hospital of North Sichuan Medical College, Nanchong, Sichuan, China

**Keywords:** colonic stenting, intestinal obstruction, left-sided obstructive colon cancer, neoadjuvant chemotherapy

## Abstract

**Background:**

The effective management of acute left-sided obstructive colon cancer (LSOCC) remains challenging. This meta-analysis compared the short-term and long-term outcomes between two prevalent treatment approaches: selective surgery following colonic stenting (CS) and colonic stenting combined with neoadjuvant chemotherapy (CS-NAC).

**Methods:**

We conducted a comprehensive literature search of databases, including PubMed and Web of Science, covering the period from January 2000 to May 2025. Relevant studies comparing CS with CS-NAC were identified and analyzed. Both the short-term and long-term outcomes of the two treatment strategies were evaluated. The meta-analysis calculated pooled odds ratios (ORs), mean differences (MDs), and 95% confidence intervals (95% CIs). This study is registered with PROSPERO (registration number: CRD42024580176).

**Results:**

Seven studies were included in the meta-analysis. Compared with the CS strategy, the CS-NAC strategy demonstrated significantly better short-term outcomes, including higher rates of laparoscopic surgery, lower intraoperative stoma rates, fewer overall postoperative adverse events, shorter operative times, and shorter postoperative hospital stays. However, no significant differences were observed between the two strategies in terms of stent-related complications or specific short-term adverse events, including postoperative incision infection rates, anastomotic leakage rates, and pulmonary infection rates. Furthermore, compared with the CS strategy, the CS-NAC strategy significantly improved both 3-year disease-free survival and overall survival.

**Conclusion:**

Compared with CS alone, the CS-NAC treatment strategy improves laparoscopic surgery rates and 3-year survival outcomes without increasing the risk of certain postoperative adverse events.

## Introduction

Left-sided obstructive colon cancer (LSOCC) represents a critical clinical challenge, accounting for 70% of colorectal cancer obstructions due to anatomical narrowing of the left colon. Approximately 20% of colorectal cancer patients develop obstructions, which carry life-threatening risks without prompt intervention^[1–3]^. Compared with nonobstructive malignancies, LSOCC is associated with greater surgical risks and more aggressive biological behavior, leading to suboptimal surgical outcomes and reduced survival^[4]^. These complexities underscore the need for optimized therapeutic strategies.

Current management approaches for malignant colorectal obstruction include emergency colectomy, palliative decompression stoma, and colonic stent placement (CS). Given the inherent morbidity and mortality risk of emergency surgery and the negative impact of stoma on patients’ quality of life, the use of CS as a bridge to surgery (CS-BTS) has gained increasing attention^[5]^. While CS effectively relieves acute obstruction, it faces several challenges, including stent-related complications (SRCs) (e.g., migration, perforation), persistent intestinal wall edema, stoma rates as high as 34%, and potential oncologic risks such as tumor dissemination^[6–10]^. Emerging evidence suggests that combining neoadjuvant chemotherapy with CS (CS-NAC) may overcome these limitations^[11,12]^. Compared with CS alone, CS-NAC has potential advantages in increasing the laparoscopic surgery rate, promoting primary anastomosis, reducing overall adverse events, and improving long-term survival. These benefits may result from chemotherapy-induced tumor downstaging and resolution of intestinal edema, facilitating minimally invasive approaches and radical resection and positioning CS-NAC as a promising therapeutic strategy for LSOCC.

Despite these advances, significant knowledge gaps persist. Current evidence primarily comes from single-center studies with methodological heterogeneity regarding chemotherapy regimens, treatment durations, and outcome reporting^[13–19]^. Furthermore, no meta-analysis has systematically compared CS-NAC with CS-BTS, leaving clinicians without consensus on optimal management.

This meta-analysis addresses these uncertainties by synthesizing evidence from seven comparative studies (n = 446)^[13–19]^. We evaluated both short-term surgical outcomes and long-term survival while exploring sources of heterogeneity through subgroup analyses. Our findings aim to provide evidence-based recommendations for managing this complex patient population.

## Methods

This meta-analysis was conducted following the PICOS framework to ensure methodological rigor (Supplementary Digital Content Table S1, available at: http://links.lww.com/JS9/E667). We strictly adhered to the PRISMA 2020 Checklist^[20]^ (Supplementary Digital Content Table S2, available at: http://links.lww.com/JS9/E667), MOOSE checklist^[21]^ (Supplementary Digital Content Table S3, available at: http://links.lww.com/JS9/E667), AMSTAR-2 criteria^[22]^ (Supplementary Digital Content Table S4, available at: http://links.lww.com/JS9/E667), and the TITAN Guidelines 2025^[23]^ (Supplementary Digital Content Table S5, available at: http://links.lww.com/JS9/E667, which pertain to the declaration and use of artificial intelligence). This study protocol was registered in PROSPERO (CRD42024580176).

### Search strategy

A comprehensive systematic search was performed in the PubMed, Web of Science, Cochrane Library, Embase, CNKI, Wanfang, VIP and CBM databases along with 14 clinical trial registries from 1 January 2000 to 20 May 2025. The search terms included “colorectal neoplasms,” “neoadjuvant treatment, neoadjuvant therapy,” “intestinal obstruction,” “stents,” and their related free-text terms (Supplementary Digital Content Tables S6 and S7). All language publications are taken into account. NoteExpress 3.8 was used for citation management and duplicate removal. To minimize publication bias, we performed manual citation snowballing by screening the reference lists of the included studies and key systematic reviews^[24–27]^.

**HIGHLIGHTS**

- **Superior short-term outcomes**: Compared with CS alone, CS-NAC significantly increases laparoscopic surgery rates, reduces intraoperative stoma rates, decreases operative times, and reduces postoperative hospital stays.
- **Enhanced survival benefits**: The CS-NAC strategy significantly improves 3-year disease-free survival (DFS) and overall survival (OS) rates, offering long-term advantages over CS alone.
- **Comparable safety profile**: Despite its significant efficacy, CS-NAC does not lead to higher rates of stent-related complications such as perforation or stent migration, or higher rates of postoperative complications such as anastomotic leakage, incision infection, or pulmonary infection.
- **Subgroup analysis**: The benefits of CS-NAC in terms of reducing intraoperative stoma rates, operative times, postoperative hospital stays, and overall complications are more pronounced in subgroups of RCT studies, patients under 60 years of age, and those receiving a three-week chemotherapy regimen.

### Literature screening, quality assessment and data extraction

This meta-analysis considered reports in the form of articles, including randomized clinical trials (RCTs) and other types of controlled studies (such as cohort studies and case‒control studies), on elective surgery after CS versus elective surgery after CS-NAC. Non-RCT studies were required to use an intention-to-treat analysis. To be included in the analysis, the articles had to compare two or more treatment strategies for histologically confirmed LSCC patients with complete bowel obstruction and report at least one outcome indicator. Studies were excluded if they involved stent implantation for benign tumors, right-sided obstructive colon tumors, stent implantation for palliative treatment or other noncurative surgeries, stent placement for tumor recurrence or exogenic tumor compression, preoperative distant metastasis of the tumor, insufficient outcome variable data for the two methods, or if results could not be calculated from published data. To ensure the rigor and consistency of the studies included in this meta-analysis, only the most recent and high-quality study was selected if it shared the same database or patient cohort, was authored by the same group, and presented similar outcome indicators. Additional studies were taken into account only when they offered exclusive analyses or disclosed divergent outcomes. Furthermore, attention was given to the funding sources of the included studies.

To assess the quality of the RCTs, this study adhered to the standards of the Cochrane Handbook^[28]^. For non-RCT studies, we employed the Newcastle‒Ottawa Scale (NOS) for quality assessment^[29]^. The NOS is a recognized standardized tool specifically designed for evaluating the methodological quality of observational studies in systematic reviews and meta-analyses. The Grades of Recommendation, Assessment, Development and Evaluation (GRADE) system was utilized to rigorously evaluate the quality and certainty of all evidence included in the studies.

Data extraction was conducted via a predefined extraction form, which included baseline demographic data (age, sex), short-term outcome measures (rate of laparoscopic surgery, intraoperative stoma rate, operative time, postoperative hospital stay), short-term adverse events (postoperative incisional infection rate, anastomotic leakage rate, pulmonary infection rate, overall postoperative adverse event rate), SRCs (perforation, stent migration, obstruction, dislodgement), and long-term outcome measures (3-year disease-free survival (DFS), 3-year overall survival (OS)). All data were extracted whenever available in the studies.

Article screening, quality assessment, and data extraction were independently conducted by two researchers to ensure the rigor of the process. Any discrepancies that arose during the evaluation were resolved by consulting a third researcher, and the relevant articles were discussed until a consensus was reached.

### Outcome indicators

The primary outcome indicator was long-term prognosis, and the secondary outcome indicators were short-term surgical outcomes and SRCs.

### Meta-analysis and sensitivity analysis

The meta-analysis was performed via RevMan 5.4.1 software. Heterogeneity among the included studies was assessed via the Cochrane Q *P* value and the I^2^ statistic^[30]^; if heterogeneity could not be proven (I^2^ ≤ 50%, *P* > 0.1), a fixed-effects model was used; in contrast, if heterogeneity was present, a random-effects model was applied, followed by a sensitivity analysis^[31,32]^. This analysis involved gradually eliminating individual studies to identify the source of heterogeneity and to further analyze it. For dichotomous variables, odds ratios (ORs) were calculated. For continuous variables, the mean difference (MDs) was used. For survival rates (3-year DFS, 3-year OS), where original data could not be obtained, key time point data were extracted from Kaplan‒Meier curves via Engauge Digitizer 12.1 software (developed in the United States). While there is a potential for minor inaccuracies due to the digitization process, evidence from related research^[33]^ indicates that this approach is generally considered to be quite reliable. As some articles did not provide survival curves, enough data could not be obtained to calculate the hazard ratio (HR), so the OR was used.

### Subgroup analysis

In this meta-analysis, the included studies varied in terms of study design, participant characteristics, and NAC regimen, which may serve as sources of heterogeneity. To conduct a more comprehensive statistical analysis, we performed subgroup analyses to address specific questions regarding particular patient populations or study types. In the present study, subgroups were created on the basis of study design (RCT vs. non-RCT), patient age (< 60 years vs. ≥ 60 years), and NAC regimen (triweekly regimen vs. biweekly regimen), with the requirement that each outcome measure had at least five studies available. Meta-analyses were conducted for each subgroup, and the results are reported separately.

### Publication bias

Utilizing Stata 18.0, we performed Egger’s test to assess publication bias. Although a *P* value <0.05 suggests potential publication bias, we conducted additional rigorous evaluations. We applied the trim-and-fill method to enhance our analysis, thereby confirming the integrity and robustness of our research findings.

## Results

### Literature retrieval and inclusion criteria

**Figure 1.**
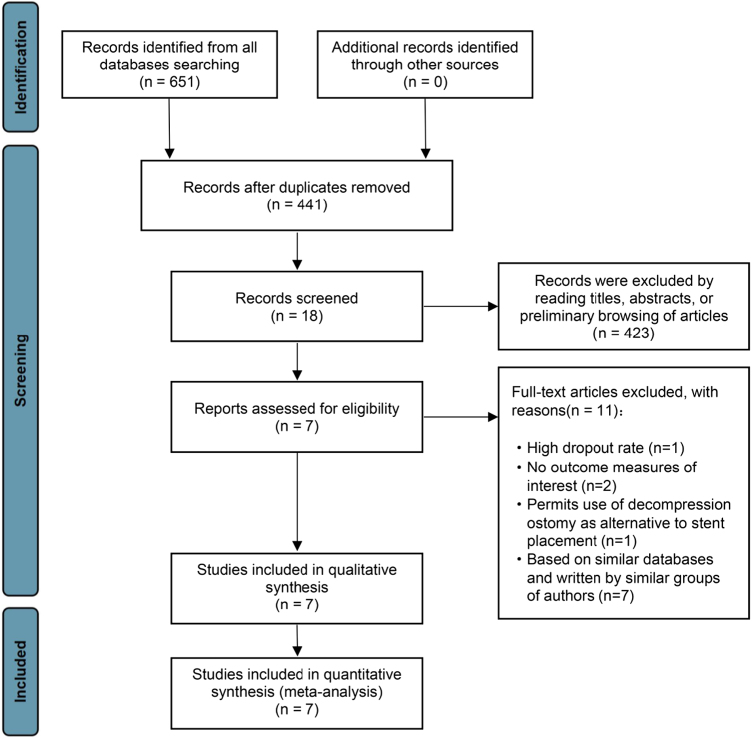
Flow diagram for literature screening.

A total of 651 articles were retrieved from the databases. After removing duplicates, 441 articles were obtained. To ensure the scientific validity of the articles, we strictly adhered to the inclusion and exclusion criteria. After initial screening through title and abstract review and full-text reading, 7 articles^[13–19]^ were ultimately included (Fig. 1), and the list of articles excluded following the full-text review can be found in Supplementary Digital Content Table S8 (available at: http://links.lww.com/JS9/E667). A total of 446 patients were included (249 in the CS group and 197 in the CS-NAC group). The basic characteristics of the included articles are shown in Table 1, the data extracted from the included studies are shown in Supplementary Digital Content Table S9 and S10 (available at: http://links.lww.com/JS9/E667) and the NAC regimens are shown in Supplementary Digital Content Table S11 (available at: http://links.lww.com/JS9/E667). The quality of the included studies is shown in Table 2. For RCTs, the quality assessment based on the Cochrane Handbook confirmed that the included trials met the research standards. For non-RCT studies, a score of 7–8 on the NOS indicates that the studies are of sufficiently high quality to be included in the meta-analysis. Except for the secondary outcome of SRCs, which was rated as “moderate” owing to high heterogeneity, the GRADE level of evidence certainty for all other outcomes was rated as “high.” This indicates that the overall quality of evidence is high and that the study results are robust (Supplementary Digital Content Table S12, available at: http://links.lww.com/JS9/E667).

**Table 1:** Basic features of the included studies

Author	Year of publication	Study design	Total sample size	CS group			CS-NAC group			Outcome indicator
				Sample size	Gender(male/female)	Age(yr)	Sample size	Gender(male/female)	Age(yr)	
Xie^[19]^	2025	Non-RCT	60	34	20/14	63.49 ± 7.56	26	18/8	62.74 ± 7.23	c,d,e,f,g,h,j
Han^[13]^	2023	Non-RCT	100	52	34/18	64.9 ± 8.8	48	31/17	64.3 ± 7.2	a,b,c,d,e,f,g,h,I,j
Zhang^[15]^	2021	Non-RCT	61	43	25/18	53.96 ± 2.51	18	10/8	54.12 ± 2.56	a,b,c,d,f,h
Cheng^[16]^	2021	RCT	70	35	18/17	51.5 ± 7.1	35	22/13	50.3 ± 7.6	d,e,f,g,h,I,j
Xu^[17]^	2019	RCT	40	20	13/7	64.7 ± 6.6	20	12/8	64.3 ± 6.2	c,e,f,g,h,j
Yin^[18]^	2019	RCT	70	35	18/17	49.56 ± 8.01	35	20/15	50.33 ± 7.78	a,b,d,e,f,g,h,I,j
Wu^[14]^	2013	Non-RCT	45	30	16/14	68.5 ± 10.2	15	9/6	67.8 ± 12.5	d

CS = elective surgery following colonic stenting, CS-NAC = colonic stenting combined with neoadjuvant chemotherapy.

^a^ 3-year disease-free survival rates.

^b^ 3-year overall survival rates.

^c^ Laparoscopic surgery rates.

^d^ Intraoperative stoma rates.

^e^ Operation time.

^f^ Postoperative hospital stay.

^g^ Postoperative incision infection rates.

^h^ Anastomotic leakage rates.

^i^ The pulmonary infection rates.

^j^ Overall postoperative adverse event rates.

**Table 2:** Evaluation of the quality of the included studies

Author	Year	Study design	Random sequence generation	Allocation concealment	Assessment of blinding	Incomplete outcome data	Selective reporting	Other bias	NOS score
Xie^[19]^	2025	Non-RCT	-	-	-	-	-	-	7
Han^[13]^	2023	Non-RCT	-	-	-	-	-	-	7
Zhang^[15]^	2021	Non-RCT	-	-	-	-	-	-	8
Cheng^[16]^	2021	RCT	High risk	Unclear	Unclear	Low risk	Low risk	Low risk	-
Xu^[17]^	2019	RCT	Low risk	Unclear	Unclear	Low risk	Low risk	Low risk	-
Yin^[18]^	2019	RCT	Low risk	Unclear	Unclear	Low risk	Low risk	Low risk	-
Wu^[14]^	2013	Non-RCT	-	-	-	-	-	-	7

### 3-year disease-free survival rates

**Figure 2.**
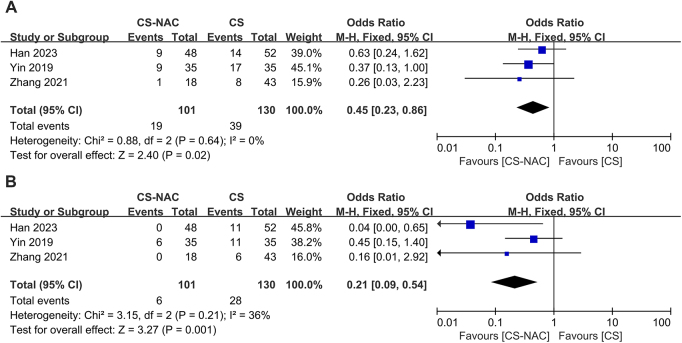
A. Forest plot of 3-year disease-free survival rates. B. Forest plot of 3-year overall survival rates.

Three papers^[13,15,18]^ reported the 3-year disease-free survival rate in the CS group versus the CS-NAC group, with no heterogeneity across studies (I^2^ = 0, *P* = 0.64). A fixed-effects model was used (OR = 0.45, 95% CI: 0.23–0.86, *P* = 0.020), suggesting that the CS-NAC treatment strategy improved the 3-year disease-free survival rate (Fig. 2A).

### 3-year overall survival rates

Three papers^[13,15,18]^ reported the 3-year overall survival rate in the CS group versus the CS-NAC group, with no heterogeneity across studies (I^2^ = 36%, *P* = 0.21). A fixed-effects model was used (OR = 0.21, 95% CI: 0.09–0.54, *P* = 0.001), suggesting that the CS-NAC treatment strategy improved the 3-year overall survival rate (Fig. 2B).

### Laparoscopic surgery rates

**Figure 3.**
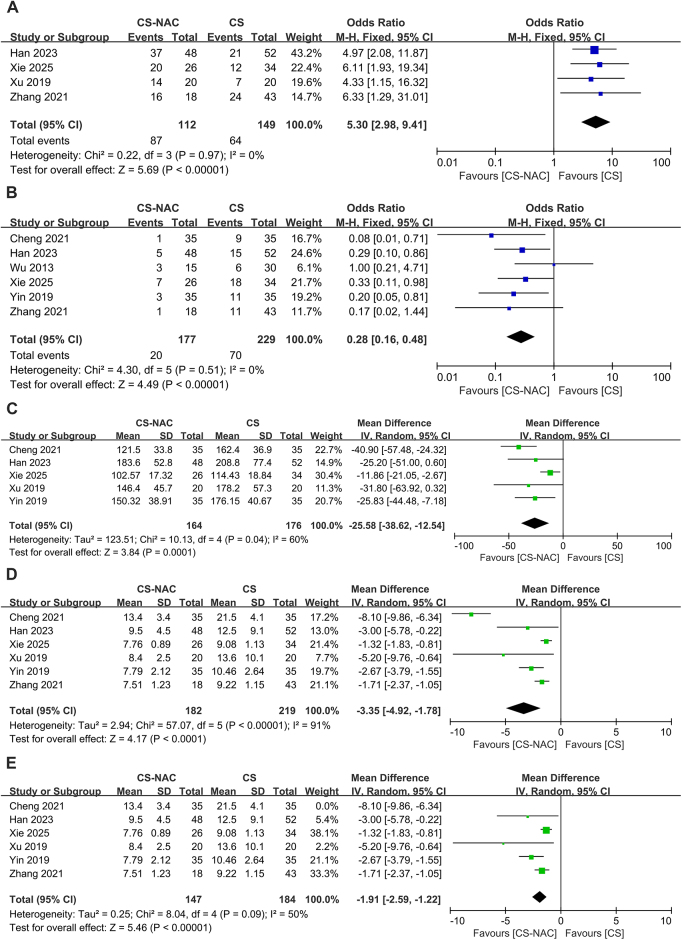
A. Forest plot of laparoscopic surgery rates. B. Forest plot of intraoperative stoma rates. C. Forest plot of operation time. D. Forest plot of postoperative hospital stay before Cheng’s article was removed. E. Forest plot of postoperative hospital stay after Cheng’s article was removed.

Four papers^[13,15,17,19]^ reported the rate of laparoscopic surgery in the CS group versus the CS-NAC group, and there was no heterogeneity among the studies (I^2^ = 0, *P* = 0.97). A fixed-effects model was used (OR = 5.30, 95% CI: 2.98–9.41, *P* < 0.000), which suggested that CS-NAC followed by elective surgery could increase the laparoscopic surgery rate (Fig. 3A).

### Intraoperative stoma rates

Six articles^[13,15–19]^ reported the intraoperative stoma rate in the CS group versus the CS-NAC group, with no heterogeneity across studies (I^2^ = 0, *P* = 0.51). A fixed-effects model was used (OR = 0.28, 95% CI: 0.16–0.48, *P* < 0.000), suggesting that the CS-NAC treatment strategy could reduce the rate of intraoperative stoma (Fig. 3B).

### Operation time

Five papers^[13,16–19]^ reported the operation time in the CS group versus the CS-NAC group, with moderate heterogeneity across studies (I^2^ = 60%, *P* = 0.04); thus, a random effects model was used (MD = −25.58, 95% CI: −38.62 to 12.54, *P* < 0.000), indicating that the CS-NAC treatment strategy can reduce the operative time (Fig. 3C). After sensitivity analysis and sequential exclusion of articles, no single article was found to significantly reduce heterogeneity when it was excluded. Subgroup analysis will be conducted to identify the sources of heterogeneity.

### Postoperative hospital stay (POHS)

Six articles^[13,15–19]^ reported POHS in the CS group versus the CS-NAC group, with high heterogeneity observed among the studies, for which a random effects model was used (I^2^ = 91%, *P* < 0.00001) (Fig. 3D). After sensitivity analysis and sequential exclusion of articles, the exclusion of the article by Cheng *et al*^[16]^. The heterogeneity was significantly reduced to a moderate level (I^2^ = 50%, *P* = 0.09), but further subgroup analysis is still needed. A random-effects model was used (MD = −1.91, 95% CI: −2.59 to −1.22, *P* < 0.000), indicating that the CS-NAC treatment strategy can shorten the POHS (Fig. 3E).

### Incidence of postoperative adverse events

**Figure 4.**
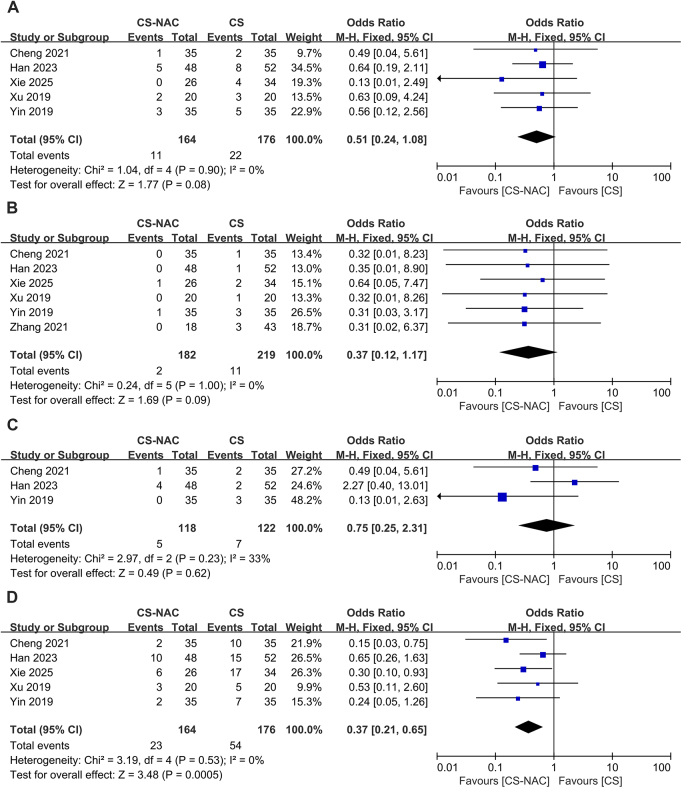
A. Forest plot of postoperative incision infection rates. B. Forest plot of anastomotic leakage rates. C. Forest plot of pulmonary infection rates. D. Forest plot of overall postoperative adverse event rates.

Six articles^[13,15–19]^ reported the incidence of postoperative adverse events in the CS group versus the CS-NAC group, of which incision infection, anastomotic leakage, and pulmonary infection were more common. Five papers^[13,16–19]^ reported the postoperative incision infection rates in the CS group and the CS-NAC group. There was no heterogeneity in the studies (I^2^ = 0, *P* = 0.90), and a fixed-effects model was used (OR = 0.51, 95% CI: 0.24–1.08, *P* = 0.080), which suggested that the rate of postoperative incisional infection was similar between the two groups (Fig. 4A). The anastomotic leakage rate in the CS group versus the CS-NAC group was reported in six papers,^[13,15–19]^ with no heterogeneity among studies (I^2^ = 0, *P* = 1.00), and according to a fixed-effects model (OR = 0.37, 95% CI: 0.12–1.17, *P* = 0.090), there was no significant difference in the rate of anastomotic leakage between the two groups (Fig. 4B). Three papers^[13,16,18]^ reported the pulmonary infection rate in the CS group versus the CS-NAC group, with good homogeneity among the studies (I^2^ = 33%, *P* = 0.23), and a fixed-effects model (OR = 0.75, 95% CI: 0.25–2.31, *P* = 0.620) was used; it was concluded that the pulmonary infection rates were similar in both groups (Fig. 4C). Five papers^[13,16–19]^ reported the overall postoperative adverse event rate in the CS group versus the CS-NAC group, with no heterogeneity among the studies (I^2^ = 0, *P* = 0.53), and a fixed-effects model (OR = 0.37, 95% CI: 0.21–0.65, *P* < 0.001) was used, indicating that the CS-NAC treatment strategy reduced the overall postoperative adverse event rate (Fig. 4D).

### SRCs

**Figure 5.**
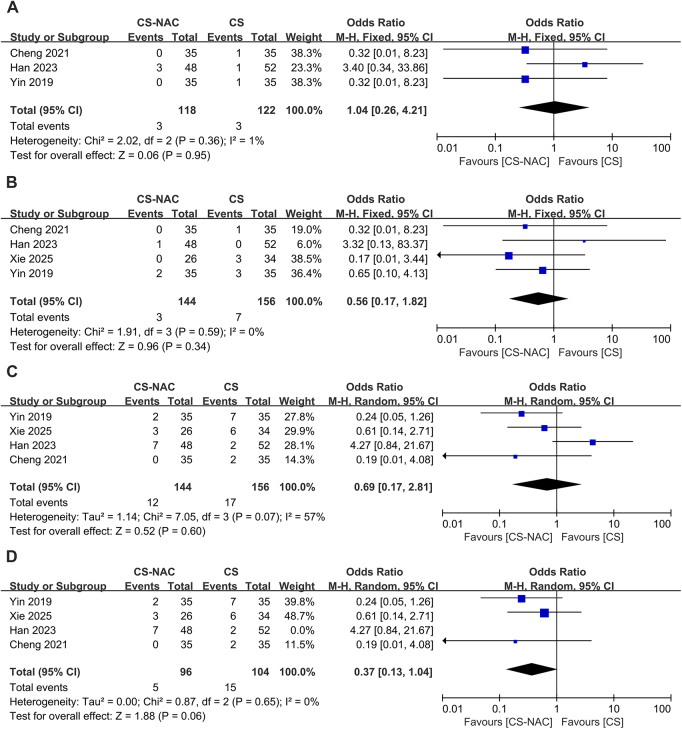
A. Forest plot of perforation rates. B. Forest plot of the stent migration rates. C. Forest plot of the incidence of major stent-related complications before the exclusion of Han’s article. D. Forest plot of the incidence of major stent-related complications after the exclusion of Han’s article.

Four articles^[13,16,18,19]^ reported the incidence of SRCs in the CS group and the CS-NAC group. Among them, three articles^[13,16,18]^ reported perforation rates and showed no heterogeneity (I^2^ = 1%, *P* = 0.36), and a fixed-effects model was used (OR = 1.04, 95% CI: 0.26–4.21, *P* = 0.950), which indicated that the perforation rate was similar between the two groups (Fig. 5A). Four^[13,16,18,19]^ articles reported the rate of stent migration with no heterogeneity (I^2^ = 0, *P* = 0.59). According to a fixed-effects model (OR = 0.56, 95% CI: 0.17–1.82, *P* = 0.340), there was no significant difference in the rate of stent migration between the two groups (Fig. 5B). Four articles^[13,16,18,19]^ reported the incidence of major SRCs (including perforation, migration, dislodgement, and obstruction). Given the moderate heterogeneity among the studies (I^2^ = 57%, *P* = 0.07), a random effects model was used (Fig. 5C). After sensitivity analysis and sequential exclusion of articles, the exclusion of the article by Han *et al*^[13]^ significantly reduced the heterogeneity (I^2^ = 0%, *P* = 0.65). A random-effects model was then used (OR = 0.37, 95% CI: 0.13–1.04, *P* = 0.060), indicating that the incidence of major SRCs was similar between the two groups (Fig. 5D).

### High dropout rate control

To control for the potential impact of high dropout rates on the results, we excluded the article by Han *et al*^[13]^., which contributed to a high dropout rate of 12.5% in the CS-NAC group due to SRCs (Supplementary Digital Content Figures S1–4, available at: http://links.lww.com/JS9/E667). The results revealed that there was no heterogeneity among the studies regarding 3-year DFS, 3-year OS, laparoscopic surgery rate, intraoperative stoma rate, postoperative complication rate, or SRC rate. However, heterogeneity remained for operative time and POHS, which was consistent with the findings before exclusion. Specifically, the data for 3-year DFS, the laparoscopic surgery rate, the intraoperative stoma rate, and the overall postoperative complication rate still favored the CS-NAC group over the CS group. The rates of incisional infection, anastomotic leakage, pulmonary infection, and SRCs remained statistically nonsignificant. With respect to 3-year OS, after Han’s study was excluded, the *P* value changed from 0.001 to 0.060, indicating that the difference was not statistically significant (*P* < 0.05) (Supplementary Digital Content Figure S1B, available at: http://links.lww.com/JS9/E667).

### Subgroup analysis

To further explore the impact of study design, patient age, and the NAC regimen on short-term patient outcomes, we conducted subgroup analyses (Supplementary Digital Content Figure S5–7, available at: http://links.lww.com/JS9/E667). The findings indicate that in terms of surgical duration, intraoperative stoma rates, and overall complication incidence, the CS-NAC group outperformed the CS group across different subgroups, with more pronounced advantages observed in the subgroups of RCT studies, patients under 60 years of age, and those receiving the triweekly regimen (see Table 3). However, no significant differences in the rates of anastomotic leakage or incisional infection were detected between the CS-NAC and CS groups. Compared with the CS strategy, the CS-NAC strategy significantly reduced the hospital stay across various subgroups, with more notable benefits observed in the RCT subgroup and the triweekly regimen subgroup; however, similar advantages were not observed in the age subgroup. In conjunction with the previous heterogeneity test results, the subgroup analysis suggested that heterogeneity in surgical duration and POHS may be due to differences in study design, patient age, and chemotherapy regimen.

**Table 3:** Statistical comparisons of subgroup analyses

	Subgroup					
Outcome measure	Study design		Patient age		NAC regimen	
	RCT	Non-RCT	Younger than 60 years	Older than 60 years	Triweekly regimen	Biweekly regimen
Intraoperative stoma rate (OR)	0.15	0.35	0.15	0.39	0.15	0.35
Operation time (MD)	−33.93	−13.36	−33.99	−15.09	−33.93	−13.36
Postoperative hospital stay (MD)	−2.93	−1.50	−2.08	−2.30	−2.93	−1.50
Postoperative incision infection rate (OR)	0.57	0.46	0.54	0.49	0.57	0.46
Anastomotic leakage rate (OR)	0.32	0.43	0.32	0.45	0.32	0.43
Overall postoperative adverse event rates (OR)	0.26	0.48	0.19	0.48	0.26	0.48

### Publication bias

**Figure 6.**
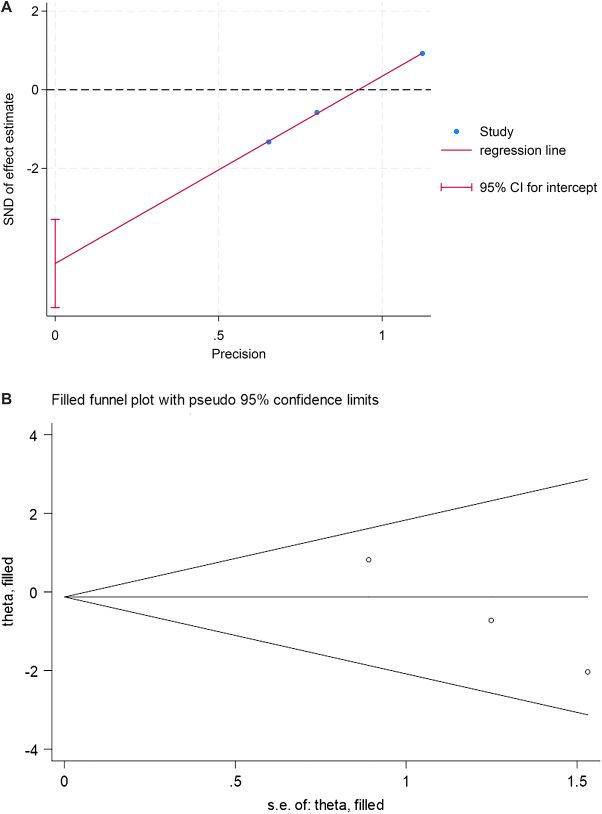
A. Egger’s test plot for pulmonary infection rates. B. Funnel plot with the trim-and-fill method for pulmonary infection rates (with an additional 0 studies imputed).

Egger’s test was used to detect publication bias. Apart from the pulmonary infection rate (*P* = 0.013), where the regression line slope was significant and the 95% CI for the intercept did not include zero, indicating the potential presence of publication bias, all other outcome measures did not demonstrate evidence of publication bias (*P* > 0.05) (**Fig.** **6A**). For the pulmonary infection rate, a trim-and-fill analysis was conducted, which revealed that the effect sizes remained unchanged before and after trimming, and the results still lacked statistical significance (**Fig.** **6B**). This finding indicates that the impact of publication bias was minimal, rendering the results robust.

## Discussion

This study, for the first time through meta-analysis, validated the significant impact of the combined strategy of CS-NAC on both short-term and long-term prognosis in patients with LSOCC. The results revealed that the CS-NAC strategy significantly improved 3-year DFS and OS in patients with LSOCC without increasing the incidence of stent- or surgery-related complications. These findings provide high-level evidence-based medical support for the sequential treatment model of LSOCC and have important clinical implications.

Since Tejero’s team^[34]^ first applied self-expanding metal stents (SEMSs) to treat LSOCC in 1994, with continuous advancements in endoscopic technology, CS-BTS has been adopted in clinical practice. Previous studies and meta-analyses have confirmed that, compared with emergency surgery, CS-BTS significantly reduces short-term mortality and postoperative complications^[35,36]^. Therefore, the NCCN guidelines recommend it as the first-line treatment for obstructive colorectal cancer. However, in clinical practice, CS-BTS has two limitations: first, intestinal edema persists for 1–2 weeks after stent placement, affecting bowel preparation quality^[13]^; second, it potentially increases the risk of recurrence (RR = 1.339), which may compromise long-term survival benefits^[5,37]^. The CS-NAC strategy can significantly improve the clinical outcomes of patients with LSOCC through a multistage synergistic effect. First, the placement of a colonic stent (CS) can rapidly relieve mechanical bowel obstruction^[38–40]^, improve a patient’s nutritional status and overall function, and provide a basis for tolerance of subsequent chemotherapy. NAC can reduce the volume of the primary tumor to further relieve obstruction^[41,42]^ and eliminate circulating tumor cells to reduce the risk of metastasis^[43–48]^. Moreover, the adequate time window during the neoadjuvant period is conducive to the full resolution of intestinal edema. In combination with mechanical bowel preparation and nutritional support, it optimizes surgical conditions, enhances the R0 resection rate, and enables more accurate lymph node dissection, thereby providing patients with long-term survival benefits^[48–50]^.

In addition to survival benefits, the CS-NAC strategy significantly optimized perioperative outcomes. Compared with CS, it increased the likelihood of laparoscopic surgery by 5.30 times (OR = 5.30), reduced the risk of intraoperative stoma formation by 72% (OR = 0.28), shortened the operative time by 25.58 minutes (MD = −25.58), reduced POHS by 1.91 days (MD = −1.91), and decreased the overall postoperative adverse event rate by 63% (OR = 0.37). Notably, there were no statistically significant differences between the two groups in terms of SRCs (reobstruction, migration, perforation, and stent dislodgement: 8.3% vs. 10.9%, *P* = 0.60), incisional infection rates (6.7% vs. 12.5%, *P* = 0.08), anastomotic leakage rates (1.1% vs. 5.0%, *P* = 0.09), or pulmonary infection rates (4.2% vs. 5.7%, *P* = 0.62). The comprehensive analysis indicates that the CS-NAC strategy not only improves short-term surgical outcomes but also does not increase the risk of SRCs or postoperative adverse events such as anastomotic leakage and incisional infection due to neoadjuvant treatment. This confirms that it achieves dual optimization in enhancing long-term survival benefits and ensuring short-term safety.

The successful implementation of CS-NAC critically depends on the technical success and quality of CS placement. Patients may withdraw from the study because of SRCs, which could compromise the accuracy of the research outcomes. To control for high dropout rates, we excluded studies with elevated attrition^[13]^. The results demonstrated consistency across all outcome measures before and after exclusion, except for the 3-year OS *P* value, which increased from 0.001 to 0.060. These findings further validate the significant clinical benefits of the CS-NAC strategy for both short- and long-term patient outcomes.

Additionally, we observed that Cheng’s study^[16]^, which utilized digital subtraction angiography (DSA)-guided CS placement (instead of traditional endoscopy) and a shortened preoperative waiting period (5–8 days, instead of the recommended 2 weeks), reported higher complication rates and served as the primary source of heterogeneity in POHS duration (I^2^ = 91% → 50%). These findings highlight that endoscopic-guided CS placement combined with adherence to a 2-week preoperative waiting period is essential for ensuring CS-NAC efficacy, underscoring the urgent need to establish standardized technical protocols.

Owing to the inherent differences in study design, patient age, and NAC regimens among the current studies, this study conducted subgroup analyses. The results revealed that the CS-NAC group significantly outperformed the CS group in terms of intraoperative stoma rates, surgical time, and overall complications across different subgroups. Moreover, the RCT studies, patients under 60 years of age, and the three-week chemotherapy regimen subgroups demonstrated more pronounced advantages in the aforementioned indicators than their respective counterparts did. However, no significant differences were observed among the subgroups in terms of anastomotic leakage rates or incisional infection rates. Compared with the CS strategy, the CS-NAC strategy significantly reduced the hospitalization time across various subgroups, with more notable benefits observed in the RCT subgroup and the three-week regimen subgroup; however, similar advantages were not observed in the age subgroup. These findings reveal that heterogeneity in surgical time and POHS may stem from differences in study design, patient age, and NAC regimens. Therefore, the potential benefits of the CS-NAC strategy for patients may have been underestimated. Hence, in specific clinical practices, the chemotherapy regimen should preferably be the least impactful three-week plan, and the CS-NAC strategy should be given priority consideration for patients under 60 years of age with LSOCC.

Additionally, all included studies avoided bevacizumab and cetuximab (TS S10), likely because concerns about antiangiogenic drugs increase the incidence of SRCs such as perforation^[51–55]^. While no direct evidence exists, clinicians must carefully balance antitumor efficacy and safety when selecting NAC regimens.

The study still has the following limitations that need to be considered: (1) The number of studies meeting the inclusion criteria and having the same outcome measures is limited, resulting in a small sample size. This may affect the reliability of the results and requires cautious interpretation. (2) The studies included in this research included both RCTs and non-RCTs. Subgroup analysis revealed that study design had a significant effect on operative time and POHS. In addition, differences across studies in terms of stent implantation techniques, time intervals between CS or CS-NAC and radical surgery, NAC regimens, and postoperative adjuvant chemotherapy regimens, as well as unmeasured and unreported confounding factors in non-RCTs (such as the experience level of surgeons and postoperative patient TNM staging), may still potentially influence the results. (3) Although the overall dropout rate in the included studies was low and intention-to-treat analysis was used, the nutritional status of the included patients and their tolerance to chemotherapy were unknown, which increases the possibility of selection bias. (4) The included literature has geographical bias, and the influence of population characteristics may reduce the generalizability and credibility of the results. (5) Potential minor errors from extracting Kaplan‒Meier curve data. However, Egger’s test and trim-and-fill analysis showed robustness for most outcomes, with the exception of slight publication bias in pulmonary infection rates.

Despite the aforementioned limitations, our research findings suggest that CS-NAC is a viable strategy for LSOCC, particularly benefiting patients under the age of 60 and those receiving a three-week chemotherapy regimen more significantly. Future large-scale, multicenter RCTs are needed to further verify its long-term efficacy and safety, especially given the current study’s geographical limitations, small sample size, and inconsistencies in pathology results and adjuvant therapy reporting.

## Conclusion

This study demonstrated that the CS-NAC strategy can significantly improve long-term survival in patients with LSOCC while maintaining good short-term safety. It can be considered a novel treatment option for LSOCC patients, with more pronounced benefits observed in those under 60 years of age and those receiving a triweekly regimen. However, given the limitations of the included studies, despite robust results, multicenter randomized trials are necessary to further validate its short- and long-term efficacy.

## Data Availability

The datasets used and analyzed during the current study are available from the corresponding author on reasonable request.

## Research registration unique identifying number (UIN)

Prospero registration number: CRD42024580176.

## AI usage statement

During the manuscript writing process, we used the generative artificial intelligence tool DeepSeek-V3 to a limited extent (accessed in May 2025), solely for the purpose of language polishing, that is, to improve grammar, syntax, and language style based on the original writing of the authors. After a comprehensive revision of the manuscript, our research team further used the AJE artificial intelligence editing service (https://china.aje.com/en/researcher/submit/get-started) to conduct a thorough review of grammar and formatting and obtain professional evaluations.

It should be emphasized that the aforementioned artificial intelligence tools were never used to generate scientific content, conduct literature searches, analyze data, or derive conclusions throughout the research process. All scientific content, research design, data processing, and interpretation of conclusions were independently completed by the author team, who are fully responsible for the integrity and accuracy of the manuscript. We are fully aware of the inherent limitations of artificial intelligence tools, and all sentences polished by AI were also strictly reviewed by the authors and finally confirmed to ensure full compliance with journal authorship and academic ethical standards. In addition, we solemnly declare that there is no conflict of interest or financial relationship with the AI provider.

## References

[1] Billingsley KG Morris AM Dominitz JA. Surgeon and hospital characteristics as predictors of major adverse outcomes following colon cancer surgery: understanding the volume-outcome relationship. Arch Surg (Chicago, Ill: 1960) 2007;142:23–31. 10.1001/archsurg.142.1.23 17224497

[2] Seo SY Kim SW. Endoscopic management of malignant colonic obstruction. Clin Endosc 2020;53:9–17. 31906606 10.5946/ce.2019.051 PMC7003005

[3] Baer C Menon R Bastawrous S Bastawrous A. Emergency presentations of colorectal cancer. Surg Clin North Am 2017;97:529–45. 28501245 10.1016/j.suc.2017.01.004

[4] Wu HW Deng W Yao HW. Clinicopathological features and prognosis analysis of the obstructive colorectal cancer Chinese. J Dig Surg 2018;17:148–53.

[5] Shang R Han X Zeng C. Colonic stent as a bridge to surgery versus emergency rection for malignant left-sided colorectal obstruction: a systematic review and meta-analysis of randomized controlled trials. Medicine 2023;102:e36078. 38115371 10.1097/MD.0000000000036078 PMC10727616

[6] Bonfante P D’Ambra L Berti S Falco E Cristoni MV Briglia R. Managing acute colorectal obstruction by “bridge stenting” to laparoscopic surgery: our experience. World J Gastrointest Surg 2012;4:289–95. 23493809 10.4240/wjgs.v4.i12.289 PMC3596526

[7] Cao K Wang ZJ Han JG. Treatment of obstructive colorectal cancer. Chinese J Gastrointest Surg 2023;26:44–50. 10.3760/cma.j.cn441530-20221114-00465 36649999

[8] Small AJ Coelho-Prabhu N Baron TH. Endoscopic placement of self-expandable metal stents for malignant colonic obstruction: long-term outcomes and complication factors. Gastrointest Endosc 2010;71:560–72. 20189515 10.1016/j.gie.2009.10.012

[9] Sloothaak DA van den Berg MW Dijkgraaf MG. Oncological outcome of malignant colonic obstruction in the Dutch Stent-In 2 trial. Br J Surg 2014;101:1751–57. 25298250 10.1002/bjs.9645

[10] Pavlidis ET Galanis IN Pavlidis TE. Management of obstructed colorectal carcinoma in an emergency setting: an update. World J Gastrointest Oncol 2024;16:598–613. 38577464 10.4251/wjgo.v16.i3.598 PMC10989363

[11] Nakagawa K Ishibe A Ohya H. Effects of neoadjuvant chemotherapy for patients with obstructive colon cancer: a multicenter propensity score-matched analysis (YCOG2101). Ann Gastroenterol Surg 2024;8:262–72. 38455492 10.1002/ags3.12736 PMC10914701

[12] Liu Y Liu P Wu MM Wei GH Wang ZJ Liu ZX. Effect of endoscopic stent placement combined with neoadjuvant chemotherapy on short-term and long-term results in patients with acute left-sided malignant colorectal obstruction without distant metastases. Zhonghua Yi Xue Za Zhi 2019;99:2348–54. 31434415 10.3760/cma.j.issn.0376-2491.2019.30.006

[13] Han JG Wang ZJ Dai Y. Short-term outcomes of elective surgery following self-expandable metallic stent and neoadjuvant chemotherapy in patients with left-sided colon cancer obstruction. Dis Colon Rectum 2023;66:1319–28. 35671281 10.1097/DCR.0000000000002372

[14] Wu J Rong DQ Liu QF. Endoscopic stenting combined with neoadjuvant chemotherapy for treatment of malignant colorectal obstruction. World Chin J Digestol 2013;21:4056–59.

[15] Zhang Z Zhuang BR Chen C. Surgical safety and medium and long term efficacy of colon stent combined with neoadjuvant chemotherapy in patients with left colon cancer complicated with obstruction. J Med Theory Pract 2021;34:4288–90.

[16] Cheng SJ Yang YM Yuan B Lu JL Deng C. Selective left hemicolectomy after expandable stent placement to relieve obstruction combined with neoadjuvant chemotherapy in the treatment of obstructive left colon cancer. Chin J Oper Surg Gen Surg 2021;15:430–33.

[17] Xu MC Feng JM Lin ZW. Clinical observation of endoscopic stent implantation combined with neoadjuvant chemotherapy in the treatment of obstructive left hemicolon carcinoma. Hainan Med J 2019;30:2079–81.

[18] Yin XQ Liu J. The efficacy of stent implantation combined with neoadjuvant chemotherapy in complete left-sided colonic cancer obstruction: a clinical trial of 70 patients. J. Colorectal & Anal Surg 2019;25:298–302.

[19] Xie DJ Sun XD Zhang XP, and Wang XZ. Curative effect and safety of metal stent combined with neoadjuvant chemotherapy in obstructive colon cancer. Jilin Med J 2025;46:798–802.

[20] Page MJ McKenzie JE Bossuyt PM. The PRISMA 2020 statement: an updated guideline for reporting systematic reviews. BMJ (Clinical Research Ed) 2021;372:n71. 10.1136/bmj.n71 PMC8005924 33782057

[21] Stroup DF Berlin JA Morton SC. Meta-analysis of observational studies in epidemiology: a proposal for reporting. Meta-analysis Of Observational Studies in Epidemiology (MOOSE) group. Jama 2000;283:2008–12. 10789670 10.1001/jama.283.15.2008

[22] Shea BJ Reeves BC Wells G. AMSTAR 2: a critical appraisal tool for systematic reviews that include randomised or non-randomised studies of healthcare interventions, or both. BMJ (Clinical Research Ed) 2017;358:j4008. 10.1136/bmj.j4008 PMC5833365 28935701

[23] Agha RA Mathew G Rashid R. Transparency In The reporting of Artificial INtelligence – the TITAN guideline. Prem J Sci 2025;10:100082.

[24] Spannenburg L Sanchez Gonzalez M Brooks A. Surgical outcomes of colonic stents as a bridge to surgery versus emergency surgery for malignant colorectal obstruction: a systematic review and meta-analysis of high quality prospective and randomised controlled trials. Eur J Surg Oncol 2020;46:1404–14. 32418754 10.1016/j.ejso.2020.04.052

[25] McKechnie T Springer JE Cloutier Z. Management of left-sided malignant colorectal obstructions with curative intent: a network meta-analysis. Surg Endosc 2023;37:4159–78. 36869265 10.1007/s00464-023-09929-4 PMC9984133

[26] Gavriilidis P De’angelis N Wheeler J Askari A Di Saverio S Davies JR. Diversion, resection, or stenting as a bridge to surgery for acute neoplastic left-sided colonic obstruction: a systematic review and network meta-analysis of studies with curative intent. Ann Royal Coll Surg Engl 2021;103:235–44. 10.1308/rcsann.2020.7137 PMC10335088 33682486

[27] Tan L Liu ZL Ran MN. Comparison of the prognosis of four different treatment strategies for acute left malignant colonic obstruction: a systematic review and network meta-analysis. World J Emerg Surg 2021;16:11. 33736680 10.1186/s13017-021-00355-2 PMC7977175

[28] Cumpston M Li T Page MJ. Updated guidance for trusted systematic reviews: a new edition of the Cochrane Handbook for Systematic Reviews of Interventions. Cochrane Database Syst Rev 2019;10:Ed000142. 31643080 10.1002/14651858.ED000142 PMC10284251

[29] Stang A. Critical evaluation of the newcastle-ottawa scale for the assessment of the quality of nonrandomized studies in meta-analyses. Eur J Epidemiol 2010;25:603–05. 20652370 10.1007/s10654-010-9491-z

[30] Higgins JP Thompson SG Deeks JJ Altman DG. Measuring inconsistency in meta-analyses. BMJ (Clinical Research Ed) 2003;327:557–60. 10.1136/bmj.327.7414.557 PMC192859 12958120

[31] Deeks JJ Higgins JPT Altman DG Chapter 10: Analysing data and undertaking meta-analyses Cochrane Handbook for Systematic Reviews of Interventions 2nd Edition Higgins JPT Thomas J Chandler J Cumpston M Li T Page MJ Welch VA (Chichester (UK): John Wiley & Sons). 2019;720.

[32] DerSimonian R Laird N. Meta-analysis in clinical trials. Control Clin Trials 1986;7:177–88. 3802833 10.1016/0197-2456(86)90046-2

[33] Jelicic Kadic A Vucic K Dosenovic S Sapunar D Puljak L. Extracting data from figures with software was faster, with higher interrater reliability than manual extraction. J Clin Epidemiol 2016;74:119–23. 26780258 10.1016/j.jclinepi.2016.01.002

[34] Tejero E Mainar A Fernández L Tobío R De Gregorio MA. New procedure for the treatment of colorectal neoplastic obstructions. Dis Colon Rectum 1994;37:1158–59. 7956588 10.1007/BF02049822

[35] Veld JV Amelung FJ Borstlap WAA. Changes in management of left-sided obstructive colon cancer: national practice and guideline implementation. J Natl Compr Canc Netw 2019;17:1512–20. 31805533 10.6004/jnccn.2019.7326

[36] Ormando VM Palma R Fugazza A Repici A. Colonic stents for malignant bowel obstruction: current status and future prospects. Expert Rev Med Devices 2019;16:1053–61. 31778081 10.1080/17434440.2019.1697229

[37] Sabbagh C Browet F Diouf M. Is stenting as “a bridge to surgery” an oncologically safe strategy for the management of acute, left-sided, malignant, colonic obstruction? A comparative study with a propensity score analysis. Ann Surg 2013;258:107–15. 23324856 10.1097/SLA.0b013e31827e30ce

[38] Gallardo-Valverde JM Calañas-Continente A Baena-Delgado E. Obstruction in patients with colorectal cancer increases morbidity and mortality in association with altered nutritional status. Nutr Cancer 2005;53:169–76. 16573378 10.1207/s15327914nc5302_6

[39] Lin YM Hegde S Cong Y Shi XZ. Mechanisms of lymphoid depletion in bowel obstruction. Front Physiol 2022;13:1005088. 36213246 10.3389/fphys.2022.1005088 PMC9533077

[40] Lauro A Binetti M Vaccari S Cervellera M Tonini V. Obstructing left-sided colonic cancer: is endoscopic stenting a bridge to surgery or a bridge to nowhere? Dig Dis Sci 2020;65:2789–99. 32583222 10.1007/s10620-020-06403-2

[41] Maas M Nelemans PJ Valentini V. Long-term outcome in patients with a pathological complete response after chemoradiation for rectal cancer: a pooled analysis of individual patient data. Lancet Oncol 2010;11:835–44. 20692872 10.1016/S1470-2045(10)70172-8

[42] Arredondo J Pastor E Simó V. Neoadjuvant chemotherapy in locally advanced colon cancer: a systematic review. Tech Coloproctol 2020;24:1001–15. 32666362 10.1007/s10151-020-02289-4

[43] Maruthachalam K Lash GE Shenton BK Horgan AF. Tumour cell dissemination following endoscopic stent insertion. Br J Surg 2007;94:1151–54. 17541987 10.1002/bjs.5790

[44] Malgras B Brullé L Lo Dico R. Insertion of a stent in obstructive colon cancer can induce a metastatic process in an experimental murine model. Ann Surg Oncol 2015;22:S1475–80. 25956578 10.1245/s10434-015-4588-y

[45] Pattarajierapan S Sukphol N Junmitsakul K Khomvilai S. Oncologic safety of colonic stenting as a bridge to surgery in left-sided malignant colonic obstruction: current evidence and prospects. World J Clin Oncol 2022;13:943–56. 36618077 10.5306/wjco.v13.i12.943 PMC9813833

[46] Yamashita S Tanemura M Sawada G. Impact of endoscopic stent insertion on detection of viable circulating tumor cells from obstructive colorectal cancer. Oncol Lett 2018;15:400–06. 29391884 10.3892/ol.2017.7339 PMC5769419

[47] Takahashi G Yamada T Iwai T. Oncological assessment of stent placement for obstructive colorectal cancer from circulating cell-free DNA and circulating tumor DNA dynamics. Ann Surg Oncol 2018;25:737–44. 10.1245/s10434-017-6300-x 29235008

[48] Foxtrot Collaborative G. Feasibility of preoperative chemotherapy for locally advanced, operable colon cancer: the pilot phase of a randomised controlled trial. Lancet Oncol 2012;13:1152–60. 23017669 10.1016/S1470-2045(12)70348-0 PMC3488188

[49] Engstrom PF Arnoletti JP Benson AB. NCCN clinical practice guidelines in oncology: colon cancer. J Natl Compr Canc Netw 2009;7:778–831. 19755046 10.6004/jnccn.2009.0056

[50] Habr-Gama A São Julião GP Perez RO. Nonoperative management of rectal cancer: identifying the ideal patients. Hematol Oncol Clin North Am 2015;29:135–51. 25475576 10.1016/j.hoc.2014.09.004

[51] Hapani S Chu D Wu S. Risk of gastrointestinal perforation in patients with cancer treated with bevacizumab: a meta-analysis. Lancet Oncol 2009;10:559–68. 19482548 10.1016/S1470-2045(09)70112-3

[52] Wichelmann TA Abdulmujeeb S Ehrenpreis ED. Bevacizumab and gastrointestinal perforations: a review from the FDA Adverse Event Reporting System (FAERS) database. Aliment Pharmacol Ther 2021;54:1290–97. 34499740 10.1111/apt.16601

[53] Storandt MH Tran NH Ehret CJ. Gastrointestinal perforation after bevacizumab: a multi-site, single-institution study with a focus on survival. World J Surg Oncol 2023;21:177. 37291587 10.1186/s12957-023-03058-x PMC10249159

[54] Qi WX Shen Z Tang LN Yao Y. Bevacizumab increases the risk of gastrointestinal perforation in cancer patients: a meta-analysis with a focus on different subgroups. Eur J Clin Pharmacol 2014;70:893–906. 24858820 10.1007/s00228-014-1687-9

[55] van Halsema EE van Hooft JE Small AJ. Perforation in colorectal stenting: a meta-analysis and a search for risk factors. Gastrointest Endosc 2014;79:970–82.e7. 24650852 10.1016/j.gie.2013.11.038

